# Developmental trajectory of episodic-like memory in rats

**DOI:** 10.3389/fnbeh.2022.969871

**Published:** 2022-11-29

**Authors:** Antonis Asiminas, Stephanie A. Lyon, Rosamund F. Langston, Emma R. Wood

**Affiliations:** ^1^Centre for Discovery Brain Sciences, University of Edinburgh, Edinburgh, United Kingdom; ^2^Simons Initiative for the Developing Brain, University of Edinburgh, Edinburgh, United Kingdom; ^3^Patrick Wild Centre, University of Edinburgh, Edinburgh, United Kingdom; ^4^Center for Translational Neuromedicine, University of Copenhagen, Copenhagen, Denmark; ^5^Cellular and Systems Medicine, Ninewells Hospital and Medical School, University of Dundee, Dundee, United Kingdom; ^6^Centre for Brain Development and Repair, Bengaluru, India

**Keywords:** spontaneous object exploration, object recognition, context, object-place-context, memory ontogeny

## Abstract

**Introduction:**

Episodic memory formation requires the binding of multiple associations to a coherent episodic representation, with rich detail of times, places, and contextual information. During postnatal development, the ability to recall episodic memories emerges later than other types of memory such as object recognition. However, the precise developmental trajectory of episodic memory, from weaning to adulthood has not yet been established in rats. Spontaneous object exploration tasks do not require training, and allow repeated testing of subjects, provided novel objects are used on each trial. Therefore, these tasks are ideally suited for the study of the ontogeny of episodic memory and its constituents (e.g., object, spatial, and contextual memory).

**Methods:**

In the present study, we used four spontaneous short-term object exploration tasks over two days: object (OR), object-context (OCR), object-place (OPR), and object-place-context (OPCR) recognition to characterise the ontogeny of episodic-like memory and its components in three commonly used outbred rat strains (Lister Hooded, Long Evans Hooded, and Sprague Dawley).

**Results:**

In longitudinal studies starting at 3–4 weeks of age, we observed that short term memory for objects was already present at the earliest time point we tested, indicating that it is established before the end of the third week of life (consistent with several other reports). Object-context memory developed during the fifth week of life, while both object-in-place and the episodic-like object-place-context memory developed around the seventh postnatal week. To control for the effects of previous experience in the development of associative memory, we confirmed these developmental trajectories using a cross-sectional protocol.

**Discussion:**

Our work provides robust evidence for different developmental trajectories of recognition memory in rats depending on the content and/or complexity of the associations and emphasises the utility of spontaneous object exploration tasks to assess the ontogeny of memory systems with high temporal resolution.

## Introduction

Episodic memory relies on the coordination of many brain regions that bind multiple memory traces into a coherent spatiotemporal episode (Eichenbaum, 2017). Due to the complexity of neurophysiological processes that underlie it, episodic memory is particularly susceptible to disruptions due to normal ageing, traumatic brain injury as well as virtually all major neurological, neuropsychiatric, neurodevelopmental, and neurodegenerative diseases (Dickerson and Eichenbaum, 2010; Souchay et al., 2013; Vakil et al., 2019). Given the potential important diagnostic and translational value of episodic memory, it is important to study the neural processes that underlie it and its ontogeny.

Understanding when and how episodic memory develops is critical for disentangling the effect of interactions between genetics and experience on the neural circuits and cognitive processes that support it. It is also important for gaining insights into developmental disease progression, and for discovering developmental windows suitable for therapeutic interventions (Guillery-Girard et al., 2013; Souchay et al., 2013; Asiminas et al., 2019). In humans, episodic memory emerges relatively late during juvenile development in comparison to other forms of memory (Gogtay et al., 2004; Guillery-Girard et al., 2013; Mullally and Maguire, 2014; Riggins et al., 2020; Ngo et al., 2021). While children as young as 4 years old are able to retrieve multi-element events, associative memory that is dependent on context discrimination appears to follow a more protracted developmental trajectory (Ngo et al., 2021), which may be connected to late development of prefrontal cortex (Giedd et al., 1999; Gogtay et al., 2004; Eichenbaum, 2017).

The circuitry underlying episodic memory has been studied extensively in rodents, both in the context of basic science as well as a vehicle for understanding the pathophysiology of neurodegenerative and neurodevelopmental disorders (Day et al., 2003; Eacott and Norman, 2004; Good et al., 2007; Langston and Wood, 2009; Davis et al., 2013; Till et al., 2015; Chao et al., 2016, 2020; Asiminas et al., 2019; Barker and Warburton, 2020). A variety of tasks have been used to assess neural mechanisms of episodic memory, including both spontaneous exploration tasks (Eacott and Norman, 2004; Langston and Wood, 2009; Chao et al., 2016; Barker and Warburton, 2020) and rule-based rewarded tasks (e.g., Day et al., 2003; Ergorul and Eichenbaum, 2004; Crystal and Smith, 2014). Using spontaneous object exploration tasks different configurations of objects, object position, contexts, and temporal order permit testing of different components of episodic-like memory. As episodic memory formation involves the binding of memory traces for what happened during a specific experience together with the spatial and temporal context in which it occurred, it has been argued that spontaneous object exploration tasks that requiring binding of objects (what), with specific locations (where) and contexts (which occasion) provide a valid model of episodic or episodic-like memory in rodents (Eacott and Norman, 2004; Davis et al., 2013; Ross and Easton, 2021).

Key advantages of spontaneous object exploration tasks, compared to food-rewarded tasks, are that they are based on one-trial learning, and therefore permit testing within acute time windows, and they do not require training that can shape subsequent behaviour of subjects. This is crucial when studying the developmental trajectory of episodic-like memory longitudinally.

Rats have been the rodent model of choice when studying the development of neural circuits that support memory processes (Langston et al., 2010; Wills et al., 2010; Ainge and Langston, 2012; Muessig et al., 2016; Shan et al., 2022). Moreover, genetic rat models are currently making unique contributions in our understanding of the pathophysiology associated with cognitive phenotypes in neurodevelopmental disorders (Till et al., 2015; Asiminas et al., 2019; Marshall et al., 2021). Therefore it is essential to determine the normal developmental trajectory of episodic-like memory in rats, and reconcile this trajectory with the development of neural circuits that are known to support it, in order to provide a basis for comparison with developmental trajectories of episodic-like memory in rat models of neurodevelopmental conditions (Cruz-Sanchez et al., 2020). Given the variety of outbred rat strains currently used, it is also important to test more than one rat strain to account for strain-specific trajectories (Andrews et al., 1995; Clemens et al., 2014; Kumar et al., 2015).

Over the last two decades, several studies have focussed on the ontogeny of various type of object memory in rats (Ainge and Langston, 2012; Westbrook et al., 2014; Ramsaran et al., 2016a,b; Travaglia et al., 2018; Cruz-Sanchez et al., 2020; Sanders et al., 2020). Overall, these studies agree that the ability of rats to exhibit memory for objects bound to other contextual and/or spatial information emerges later than the memory of objects. However, small methodological differences and/or rat strains makes interpretation of these results challenging.

In the present set of studies, we examined the development of episodic-like object-place-context memory, as well as object memory, object-context memory and object-place memory in three commonly used outbred rat strains: two hooded strains [Long Evans Hooded (LEH) and Lister Hooded (LH)], and one albino strain [Sprague Dawley (SD)]. Together with Wistar rats, these strains represent 95.7% of rat strains used in neuropsychiatric experiments (Noori et al., 2018). Using a longitudinal study design, we first explored the developmental trajectory in these four tasks in LEH and SD rats. Given the different overlapping brain circuits supporting memory in each of these tasks, we predicted that rats would exhibit distinct developmental trajectories across the tasks, but that these trajectories would be similar across strains. To control for the possibility that object memory interference and/or contextual habituation across the course of the longitudinal experiment influences the performance of the rats, we also conducted a cross-sectional study, where different rats were used as subjects at each time point. This was conducted with LH rats, which also allowed us to explore developmental trajectories in a third rat strain.

## Materials and methods

### Animals

Rats used in all studies were bred in-house and kept on a 12 h light/dark cycle (lights on: 7 a.m.; lights off: 7 p.m.). Adult rat breeding pairs were either purchased from Charles River (LH) or bred in-house (SD, LEH: University of Edinburgh). Litters were culled to eight pups shortly after birth to reduce variance due to unequal maternal attention [except from three litters in the cross-sectional study used in age points P25/26 (10 rats), P31/32 (11 rats), P45/46 (9 rats)]. If the litter was born during the day (between 8 a.m. and 5 p.m.) then that day was taken as postnatal Day 0 (P0), and if the litter was born overnight then the following day was taken as P0. Pups were weaned at P21 and were then kept in same sex groups of 2–5 rats per cage.

For the longitudinal studies with Sprague-Dawley (SD) [*n* = 16 from seven litters (1–5 rats per litter)] and Long-Evans Hooded (LEH) rats [*n* = 13 from seven litters (1–3 rats per litter)], the same male rats were used for all testing points. For the cross-sectional study with Lister Hooded (LH) rats [*n* = 173 from a total of 23 litters (8–11 rats per litter)], male and female rats from a given litter were all assigned to the same testing age group. The choice of testing point was done in a pseudo-random fashion. For details of rats, litters, and testing time points see Supplementary Table 1. All animals had unrestricted access to food and water at all times. All animal experiments were approved by the University of Edinburgh or University of Dundee Animal Welfare and Ethical Review Board before their start and were performed in accordance with the guidelines established by European Community Council Directive 2010/63/EU (22 September 2010) and with the Animal Care (Scientific Procedures) Act 1986.

### Behavioural tasks

Data collection took place across two labs. The longitudinal datasets from SD and LEH rats were collected at the University of Edinburgh (Wood lab) while the cross-sectional datasets from LH rats were collected at the University of Dundee (Langston lab).

#### Apparatus and objects

For studies conducted in the Wood lab, animals were tested in a rectangular polycarbonate testing box (76 cm long × 45 cm wide × 60 cm tall) with removable wall and floor inserts that could be rearranged to form two distinct contexts. Context 1 had wooden walls covered with white textured wallpaper and a wood-effect linoleum floor. Context 2 had matt blue painted walls and a black rubber-textured floor. The box remained in the same location within the room for both context configurations. Two 3M Dual-Lock resealable fasteners were attached to the floor, 9 cm from the box walls at north-east and north-west locations, used to keep the two objects firmly attached to the floor in the same locations for every trial. The testing box was situated on a table surrounded on three sides by a black curtain, with one opening at the south side of the box (where subjects were always placed). The distance between the curtains and east and west walls of the testing apparatus was approximately 30 cm. The north wall of the testing apparatus was immediately adjacent to the curtain. Inside the curtained enclosure a lamp situated at the north-east side of the enclosure provided additional light. A multicoloured feather duster just above the north-west corner and a high contrast 3D shopping bag just above the north-east corner provided prominent visual three-dimensional cues; these were hung just above the box but were out of reach of the subjects. These cues remained in the same position and orientation throughout the experiments regardless of which context was being used. The rest of the external environment was also kept as consistent as possible, and a radio on low volume was used to mask potentially distracting noises. An opaque holding bucket (30 cm diameter, 40 cm tall) with bedding inside, which was used to hold rats between trial phases, was placed outside the curtained environment. An overhead black and white camera was used to monitor the rat in the testing box. The video signal was fed into a DVD recorder and a computer on the desk of the experimenter, which was 2 m away from the testing box. A schematic of the arrangement of the room, curtains and testing box is depicted in Supplementary Figure 1A.

For studies conducted in the Langston lab, testing was carried out within a rectangular polycarbonate testing box (58 cm long by 40 cm wide by 47 cm tall) with a wood-effect linoleum floor. The testing box was situated in the corner of the experimental room where it remained throughout all testing procedures. The testing box could be configured to make two different contexts. Context 1 had blue walls with a black perforated rubber mat floor, whereas context 2 had white and black walls with a white plastic grid placed on the linoleum floor. The arena sat on a bench 65 cm above the ground in the corner of the room. A red plastic flower and a large green playing block were used as prominent visual three-dimensional cues and were placed in the north-east and north-west corners of the arena, suspended 40 cm above the arena floor (Supplementary Figure 2C). These cues were constantly present irrespective of the contextual configuration of the arena. An opaque holding bucket was placed next to the testing box. The overhead camera was connected to a recording device and computer at the opposite side of the room to the testing box, where the experimenter scored rat object exploration. Supplementary Figure 1B shows the arrangement of the testing room, while Supplementary Figure 1C provides photographs of the two context configurations and the prominent cues used in the Langston lab.

A variety of objects were used, which were between 8 cm × 8 cm × 8 cm and 11 cm × 11 cm × 11 cm. The objects were non-porous and could be easily cleaned (photographs of all objects are shown in Supplementary Figure 2). Each object was paired with another that differed in shape, material, colour, or texture. Analysis of the sample phase explorations pooled across all rats, tasks and time points from the two longitudinal studies confirmed that rats showed similar innate interest to both objects within each pair (Supplementary Figure 3 and Supplementary Table 5). For longitudinal studies, each object-pair was used only once per animal. For the cross-sectional study, the same four object-pairs were used for a given task across all age time points.

#### Experimental timeline

For the longitudinal study in SD rats, animals were handled in the animal facility for 6 days while still in the cage with their mothers (P16–P21). After weaning they were handled for one day (P22), in the experimental room, such that they received a total of 7 days of handling. Habituation (see below) took place on P23&P24. Behavioural testing (see below) took place on the following pairs of adjacent days: P25&P26, P32&P33, P37&P38, P43&P44, P49&P50, P55&P56, P61&P62, P70&P71.

For the longitudinal study in LEH rats, animals were handled in the animal facility for three days while still in the cage with their mothers (P19–P21). After weaning they were handled for three days in the animal facility (P22–P24) and for one day in the experimental room (P25) to reach a total of 7 days of handling. Habituation took place on P26&P27. Behavioural testing took place on the following pairs of adjacent days: P28&P29, P35&P36, P42&P43, P49&P50, P55&P56, P64&P65.

For the cross-sectional study in LH rats, animals were handled for the 7 days immediately prior to habituation and habituation took place during the 2 days before each testing point. For example, rats tested at the first testing point (P25&P26), were handled and habituated on the same time frame as the SD rats in the longitudinal study, while rats in the second testing point (P31&P32) were handled for 7 days from P22–P28 and habituated on P29&P30. Behavioural testing took place on the following pairs of adjacent days: P25&P26, P31&P32, P33&P34, P34&P35, P38&P39, P42&P43, P45&P46, P47&P48, P50&P51, P70&P71.

#### Handling and habituation procedures

Handling involved 10 min per day of gently lifting the animals multiple times and allowing them to sit on the experimenters’ arms and lap. This allowed rats to get comfortable with the experimenter and the process of being lifted from their home cage. Habituation was performed in the testing box to familiarize the animals to both contextual configurations of the testing box, to the box’s location within the stable environment, to the holding bucket that was used during the task. On the morning of the first day of habituation, the animals were placed in each context configuration in cage groups (30 min per context). In the afternoon they were placed individually into each context configuration (10 min per context). Between exposures to context 1 and context 2, rats were placed into the holding bucket for 2 min. On the second day of habituation, animals were individually habituated twice to each context configuration (once to each in the morning and once to each in the afternoon; 10 min per context exposure) but this time, two different objects were fixed in the positions where the rats would encounter objects during testing. These objects were not used again during testing. During the habituation sessions, rats were left undisturbed to explore the contexts and objects. For the cross-sectional study, an identical habituation protocol was used for each group of animals during the two days preceding testing.

#### Testing procedures

Rats were tested for a single trial on each of four different object exploration tasks over a 2-day testing period (Day l, 8.30 a.m.–12.30 p.m.: object recognition (OR), 2.30 p.m.–6.30 p.m.: object-context recognition (OCR); Day 2, 8.30 a.m.–12.30 p.m.: object-place recognition (OPR), 2.30 p.m.–6.30 p.m.: object-place-context recognition (OPCR). Each trial of each task consists of multiple “phases”: OR and OPR each have one sample phase and one test phase, whereas OCR & OPCR each have two sample phases and one test phase.

**FIGURE 1:**
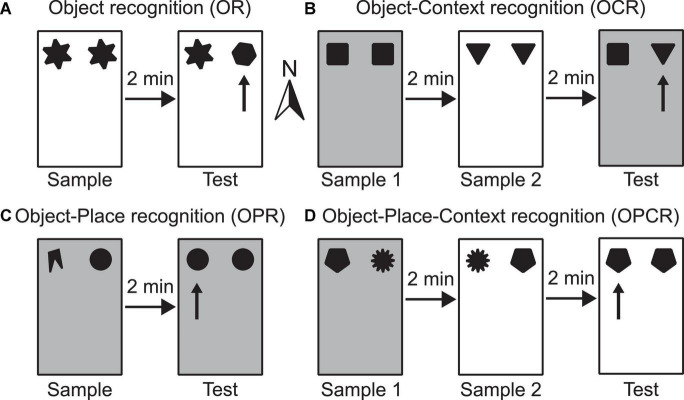
Schematic illustrations of example configurations for the four spontaneous object exploration tasks used **(A–D)**. Rectangles depict the testing box, which can be configured either as context 1 (shown as white) or context 2 (shown as grey) by changing floor and wall inserts. The different shapes represent different objects used. **(A)** OR task–the arrow indicates which object is novel in the test phase; **(B)** OCR task–the arrow indicates which objects in a novel configuration with respect to its context in the test phase; **(C)** OPR task–the arrow indicates which object is in a novel configuration with respect to its position in the test phase; **(D)** OPCR task–the arrow indicates which object is in a novel configuration with respect to the combination of position and context in the test phase. Compass arrow indicates conventional north referenced in methods’ section.

Before the start of each trial, copies of the objects needed for that trial were cleaned. For each phase of every task, the experimenter prepared the appropriate context configuration and attached two cleaned objects to the appropriate locations in the box. At the start of each phase, the rat was placed in the testing box from the south side facing the south wall of the apparatus, away from the objects (Figure 1). Prior to the first sample phase, the rat was its home cage, between phases, the rat was placed in an opaque holding bucket, and after the test phase it was returned to its home cage. During each phase, the rat was free to explore the objects and the testing box. The sample and test phases were each 3 min long and the interval between phases was 2 min. At the end of each trial and before testing the next rat, the objects and testing environment were cleaned with 70% ethanol solution and unscented baby wipes (Huggies).

Novel object positions, test phase contexts, sample phase context order (in OCR and OPCR), and identity of the object from each object pair that was designated as novel or familiar were counterbalanced across rats, tasks, and (for the longitudinal study only) time points, to ensure that the final results were as unbiased as possible. While all individual parameters were counterbalanced between rats at each time point, not all possible combinations of parameters were counterbalanced within each task at each time point. The counterbalancing overview scheme for the longitudinal testing of SD rats can be found in Supplementary Table 4. Similar counterbalancing was used for LEH longitudinal testing. For LH cross-sectional testing the same object pairs were used for every time-point/task.

#### Object recognition

The OR task consists of two phases: sample and test (Figure 1A). In the sample phase, two identical objects are available in either context 1 or context 2. In the test phase, two objects are available in the same context as the sample phase. One object is a duplicate of one of the objects used in the test phase, whereas the other is a novel object. This task is used to test whether the animal can detect object novelty and discriminate between the familiar and novel objects. Higher exploration of the novel than the familiar object is indicative of memory for the familiar object.

#### Object context recognition

The OCR task consists of three phases: sample 1, sample 2 and test (Figure 1B). In sample phase 1, two identical objects are available in either context 1 or context 2. In sample phase 2, a different pair of identical objects is available in the other context. In the test phase, two objects (one is a duplicate of the objects from sample phase 1 and the other is a duplicate of the objects from sample phase 2) are available in either context 1 or context 2. This task is used to test whether an animal can associate an object with a surrounding context. Higher exploration of the object which is in a different context than it was experienced in the sample phase is indicative of OCR memory.

#### Object place recognition

The OPR task consists of two phases: sample and test (Figure 1C). In the sample phase, two non-identical objects are available in either context 1 or context 2. In the test phase, two objects (both duplicates of one of the objects from the sample phase) are available in the same context as in the sample phase. The positions where objects are situated does not change between phases, but the association of object identity and position is. Effectively, this task is used to test whether an animal can associate a specific object with a location in space. Higher exploration of the object that is in a different location than it was experienced in the sample phase is indicative of OPR memory.

#### Object place context recognition

The OPCR tasks consists of three phases: sample 1, sample 2 and test (Figure 1D). In sample phase 1, two non-identical objects are available in either context 1 or context 2. In sample phase 2, duplicates of the same two objects used in sample phase 1 are available, but the objects have swapped locations and are in the other context. In the test phase, two identical objects (further duplicates of one of the two objects from sample phases 1 and 2) are available in one of the two contexts. This task is used to test whether the animal can associate an object with a location in a specific context. Higher exploration of the object which is in a different object-place-context configuration than it was experienced in the sample phase is indicative of OPCR memory.

### Scoring and statistical analysis

The time spent exploring each object in each sample phase and each test phase was scored manually using a simple timer computer program, with the experimenter pressing one button for each object to indicate the start and end of exploration. Object exploration was defined as the animal actively exploring an object with its snout within 2 cm of the object and performing actions such as sniffing and whisking. Exploration was not scored when the animal was not actively exploring object (e.g., climbing or resting on an object). To ensure manual scoring uniformity between experimenters and experiments, a subset of data (approximately 200 trials) were re-scored by an experimenter from the other institution (i.e., exploration originally scored “live” in Edinburgh was re-scored from video by SL at Dundee, and exploration originally scored “live” at Dundee was rescored from video by AA at Edinburgh). The re-scoring was conducted with the scorer blind to the age of the rat, to whether they were scoring a sample or a test phase, to the task that the data came from, and to which objects or object configurations were novel and familiar. Correlation of the discrimination ratios between objects calculated based on the two scorers was highly significant (R^2^ = 0.8321, *p* < 0.001).

Trials in which animals showed very low object exploration (less than 5 s of exploration of each object in a sample phase or less than 10 s of total object exploration in the test phase) were excluded from analysis. The sample sizes included in the analysis for each testing time point and each study after these exclusion criteria were applied are detailed in the figure legends and collectively shown in Supplementary Table 1 and Supplementary Table 2. The full dataset produced has been included as a supplement to the manuscript (Supplementary Table 6). For each test phase, the Discrimination Index (DI) {[(time exploring novel object or object configuration)–(time exploring familiar object or object configuration)]/(time exploring both objects)} was calculated. For all studies, one-sample *t*-tests were used to compare DIs against chance (DI = 0) controlled for the false discovery rate using the Benjamini-Hochberg procedure (Benjamini and Hochberg, 1995).

For longitudinal studies, the effects of age and strain on the total sample phase object exploration, total test phase object exploration, and the discrimination index were analyzed for each task by fitting a Linear Mixed Effects (LME) model using Maximum Likelihood (ML). Week of age (week), strain and strain × week interaction were used as fixed factors. Rat identity and litter identity were included as random factors to account for rat and litter specific effects (Bates et al., 2015; Golub and Sobin, 2020; Yu et al., 2022). For instances where the LME-ML indicated a significant strain × week interaction, two-sample *t*-tests were conducted to compare the two strains at each time point (controlled for the false discovery rate using the Benjamini-Hochberg procedure). For these analyses, data for each rat strain were binned into different approximate “weeks of age” of the rats, to circumvent the small inconsistencies in testing days between strains. Specifically, the data points between P28–P33 were defined as 4 weeks old, P35–P38 as 5 weeks old, P42–P44 as 6 weeks old, P49&P50 as 7 weeks old, P55–P57 as 8 weeks old, and P61–P65 as 9 weeks old. As only the SD rats were tested younger than 4 weeks old (P25&P26) and older than 9 weeks old (P70&P71) these data points were not included in the LME-ML analyses. For the cross-sectional study, a LME-ML model was used to examine the effect of age on the discrimination index, total sample phase exploration, and test phase object exploration, for each task. Age was set as the only fixed factor, with rat identity as random factor, and sex-within-litter identity as a nested random factor. A two-way ANOVA was used to explore the effects of sex. Rats were used as the experimental unit in all main analyses presented in this manuscript. However, we also analyzed data using a more stringent approach aiming at eliminating intra-litter statistical correlations. For the longitudinal study on SD and LEH rats, we also analyzed the data using litter as the experimental unit. One-sample *t*-tests were used to compare litter-averaged discrimination index data against chance (DI = 0). For the cross-sectional study, data were averaged across rats of the same sex and litter. One-sample *t*-tests were used to compare litter-averaged discrimination index data for each sex against chance (DI = 0). Statistical analyses were performed using GraphPad (Prism 9.3), MATLAB (version R2021b; Mathworks), and R 4.1.2 (RStudio Team, 2021). Probabilities of *p* < 0.05 were considered as significant. Data are presented as means with error bars denoting the standard error. Distributions from all studies, tasks and ages were tested for normality using Kolmogorov-Smirnov test. Almost all distributions passed the normality test (Exceptions: Longitudinal: OCR: SD-P33, OPR: SD-P62, LEH-P57, OPCR: SD-P33, LEH-P43; Cross-sectional OR: LH-P70, OPR: LH-P48, OPCR LH-P35, LH-P48). For these time points the Wilcoxon Signed Rank test was used to test difference from chance performance (DI = 0).

## Results

### Object recognition is evident at all ages tested

**FIGURE 2:**
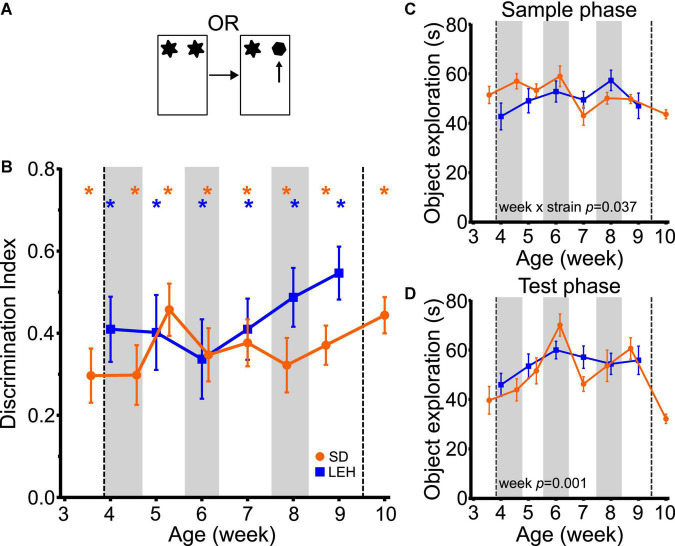
Object recognition memory is evident from the fourth week of age in LEH and SD rats. **(A)** Schematic of object recognition task; **(B)** Object recognition memory performance, expressed as a discrimination index across development. Both LEH and SD rats show significant memory from the first time tested (3rd week of age (P25) for SD and 4th week of age (P28) for LEH)–blue and orange asterisks indicate significant difference from chance (DI = 0) for LEH and SD rats respectively, based on one-sample *t*-tests. *p < 0.05. To compare directly between strains and across ages, the data were binned into 6 “week of age” bins (from ∼4 weeks to ∼9 weeks old), because the exact postnatal day of testing differed between the two strains (see section “Materials and methods” for details of bins). The different week of age bins are indicated on panels **(B–D)** with alternating vertical shading, with the first grey shaded area corresponding to 4 weeks old. As only the SD rats were tested younger than 4 weeks old (P25&P26) and older than 9 weeks old (P70&P71) these data points were not included in the cross-species comparisons (vertical dashed lines). The *p*-values for significant main effects or interactions from the LME-ML analyses between strains and weeks of age are stated within each graph. For OR memory, no significant effects of strain, week or strain × week interactions were detected; **(C)** Object exploration during sample phase for each testing time point for both LEH and SD rats. A significant week × strain interaction was detected; **(D)** Object exploration during test phase for each testing time point for both LEH and SD rats. Only a significant effect of week was detected. *p*-values from one-sample *t*-tests have been corrected for false discovery rate using the Benjamini-Hochberg procedure. [SD]: *n* = 16 for all time points; [LEH]: *n* = 13 except P35&P64 where *n* = 12. For details on sample sizes, *t*, and *p*-values for one-sample *t*-tests, see Supplementary Table 2.

Both LEH and SD rats exhibited strong preference for novel over familiar objects in the OR task at every time point, indicated by discrimination indices that were significantly higher than the chance level of zero (Figure 2B and Supplementary Figures 4A, 5A for individual animal data; one-sample *t*-tests vs. chance *p* < 0.05 for all time points in both strains). This indicates intact object recognition memory from the earliest age tested (P25 in SD, P28 in LEH) and at all subsequent ages. To compare the DIs directly between strains and across ages, the data were binned into 6 “week of age” bins (from ∼4 weeks to ∼9 weeks old), because the exact postnatal day of testing differed between the two strains (see section “Materials and methods” for details of bins). The different week of age bins are indicated on Figures 2B–D with vertical shading. A LME-ML analysis was used to compare the two strains (LEH and SD) across the different week of age bins. This revealed no significant differences between the two strains (F_(1, 27)_ = 1.948, *p* = 0.174) or between weeks (F_(5, 132)_ = 0.897, *p* = 0.485 and the strain × week interaction was also not significant (F_(5, 132)_ = 0.912, *p* = 0.476). Together, these analyses indicate stable and significant object recognition memory in SD and LEH rats from 3–4 weeks old.

We next tested whether there were differences in the total amount of object exploration in the sample and test phases across strains and week of age, as the amount of sample phase exploration can impact object recognition memory. Analysis of total sample phase object exploration revealed no significant main effects of strain (Figure 2C; LME-ML: F_(1, 27)_ = 0.763, *p* = 0.390) or week of age (F_(5, 132)_ = 1.841, *p* = 0.083), but there was a significant strain × week interaction (F_(5, 132)_ = 2.448, *p* = 0.037). However, *post-hoc* testing (multiple two sample *t*-tests with correction for multiple comparisons) did not reveal significant differences between the two strains at any age. Total test phase object exploration also did not differ between strains (Figure 2D; LME-ML: F_(1, 27)_ = 0.011, *p* = 0.916). However, we found a significant main effect of week (F_(5, 132)_ = 4.401, *p* = 0.001) with no significant strain × week interaction (F_(5, 133)_ = 1.134, *p* = 0.346).

Finally, to test whether there was an association between total sample or test phase exploration and object recognition memory, we analyzed the correlations between these variables and the discrimination index (collapsing across the different ages). This revealed a significant negative correlation between DI and both sample and test phase object exploration in SD rats (DI vs. Sample: *R* = −0.279, *p* < 0.01; DI vs. Test: *R* = −0.444, *p* < 0.001) and no significant correlations for LEH rats (Supplementary Table 3). A negative correlation between sample phase exploration and DI is surprising, as previous studies have shown that greater sample phase exploration generally leads to enhanced memory (Cohen and Stackman, 2015). Given the relatively high levels of sample phase exploration and good object recognition performance at all ages (and in both strains), we think it is unlikely that variance in sample phase exploration is influencing memory performance in the current experiment. In contrast, a negative correlation between total test phase exploration and DI might be predicted if animals explore well remembered familiar objects less than poorly remembered familiar objects. If this is the case, good memory (reflected as a higher DI) would result in lower total test phase exploration. As this relationship was only observed in the SD rats, yet object recognition memory was similar across strains, we would conclude that variability in test phase exploration is unlikely to be influencing memory performance. Together, our findings on the OR task are consistent with our previous findings as well as work from other laboratories suggesting that the ability to recognize objects emerges before the third week of life in rats (Reger et al., 2009; Ainge and Langston, 2012; Westbrook et al., 2014; Cruz-Sanchez et al., 2020).

### Object-context memory emerges around 5 weeks of age

**FIGURE 3:**
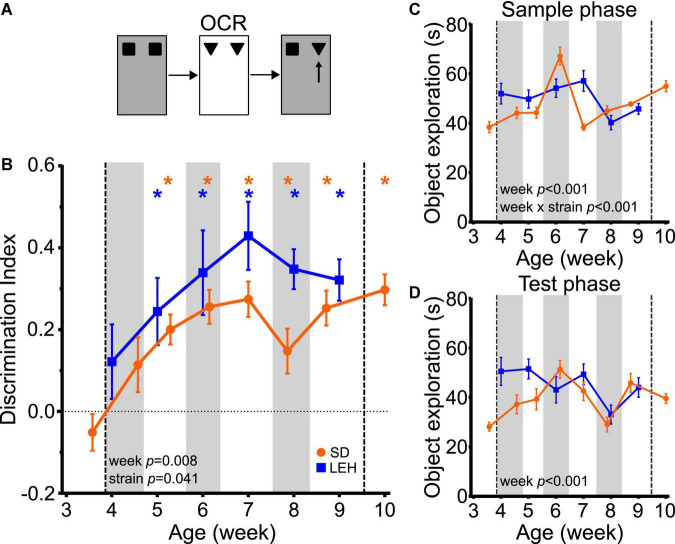
Object-context recognition memory emerges at around 5 weeks of age in LEH and SD rats. **(A)** Schematic of object-context recognition task; **(B)** Object-context recognition memory performance, expressed as a discrimination index across development. Both LEH and SD rats show significant memory at all time points from the 5th week of age (P35 for LEH, P37 for SD). *p < 0.05. Significant main effect of week and strain were found; **(C)** Object exploration during the sample phases (mean in the two sample phases) for both LEH and SD rats. A significant main effect of week and a significant week × strain interaction were detected; **(D)** Object exploration during the test phase. A significant main effect of week was detected. [SD]: *n* = 16 for all time points; [LEH]: *n* = 13 except P49 where *n* = 12. For details on sample sizes, *t*, and *p* values for one-sample *t*-tests, see Supplementary Table 2. Asterisks, shading on graphs etc., follow same convention as Figure 2.

The ability to discriminate novel from familiar object-context associations was first seen at around 5 weeks of age in both LEH and SD rats, indicated by discrimination indices that were significantly higher than the chance for all time points from five weeks old, but not at earlier time points (Figure 3B and Supplementary Figures 4B, 5B for individual animal data; one-sample *t*-tests vs. chance levels *p* < 0.05 for all time points from P35 in LEH and P37 in SD rats). Further analyses revealed a significant main effect of strain (LME-ML; F_(1, 27)_ = 4.57, *p* = 0.041) and a significant main effect of week (F_(5, 134)_ = 3.31, *p* = 0.008) but no significant strain × week interaction (F_(5, 134)_ = 0.702, *p* = 0.623). The significant difference in DI between strains reflects consistently higher discrimination ratios in LEH than in SD rats. However, given that discrimination is better than chance for both strains from week five onwards, there is no indication that the time course of development of OCR memory differs between genotypes. Rather, these analyses indicate that significant object-context recognition memory emerges sometime between 4 and 5 weeks old in both SD and LEH rats, after which it is expressed consistently.

Analysis of the total sample phase object exploration revealed no significant main effect of strain (Figure 3C; LME-ML: F_(1, 27)_ = 1.455, *p* = 0.238), but a significant main effect of week (F_(5, 134)_ = 10.528, *p* < 0.001) and a significant strain × week interaction (F_(5, 134)_ = 7.561, *p* < 0.001). *Post-hoc* tests indicated that the strains differed significantly only on week seven, where LEH rats had higher sample phase object exploration than SD rats. Analysis of the total test phase object exploration revealed no significant main effect of strain (LME-ML: F_(1, 27)_ = 3.33, *p* = 0.079), but again there was a significant main effect of week (F_(5, 134)_ = 4.419, *p* < 0.001) while the week × strain interaction was not significant (week × strain F_(5, 134)_ = 2.077, *p* = 0.072) (Figure 3D). Although both sample and test phase object exploration showed a significant main effect of week, there was no consistent trend in total object exploration over weeks for either strain, unlike the more consistent trajectory of the discrimination ratio in both strains. Correlation analyses between DI and both sample and test phase total object exploration times revealed no significant correlations for SD, and a positive correlation between DI and sample phase (but not test phase) object exploration for LEH [Supplementary Table 3; (LEH) DI vs. Sample: *R* = 0.36, *p* < 0.001]. As discussed above, more sample phase exploration could result in better memory. However, as this relationship was seen only in LEH rats, and the developmental emergence of OC recognition did not differ between strains, we think variability in sample phase exploration is unlikely to account for time course of the emergence of object-context memory. Overall, these results indicate that the ability to recognise object-context associations develops around 5 weeks of age, later than the ability to recognise objects.

### Object-place memory emerges around seven weeks of age

**FIGURE 4:**
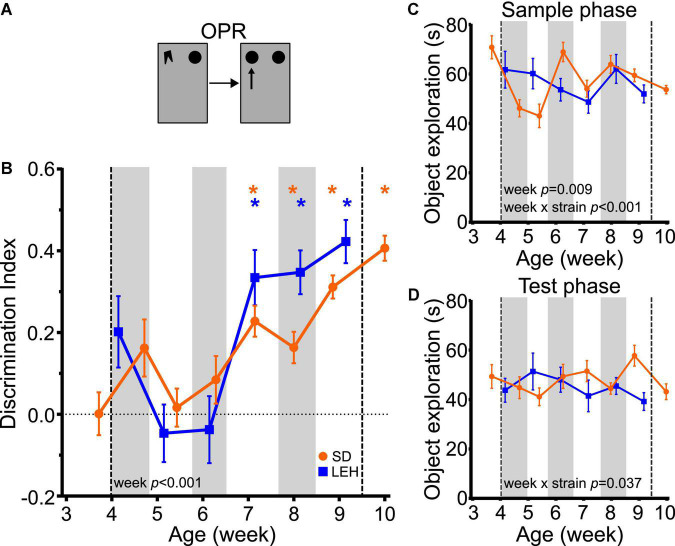
Object-place recognition memory emerges at around 7 weeks of age in LEH and SD rats. **(A)** Schematic of object-place recognition task; **(B)** Object-place recognition memory performance, expressed as discrimination index across development. Both LEH and SD rats show significant memory consistently from the 7th week of age (P50 for both LEH and SD). *p < 0.05. A significant main effect of week was found; **(C)** Object exploration during the sample and test phases for each testing time point for both LEH and SD rats. A significant main effect of week as well as a significant week × strain interaction were detected; **(D)** Object exploration during the test phase. A significant week × strain interaction was found. [SD]: *n* = 16 for all time points; [LEH]: *n* = 13 except P50 where *n* = 12. For details on sample sizes, *t*, and *p* values for one-sample *t*-tests, see Supplementary Table 2. Asterisks, shading on graphs etc., follow same convention as Figure 2.

Both SD and LEH rats can discriminate between novel and familiar object-place associations only from around 7 weeks of age, indicated by discrimination indices that were significantly higher than chance for all time points from seven weeks old, but not at earlier time points (Figure 4B and Supplementary Figures 4C, 5C for individual animal data; one-sample *t*-tests vs. chance levels *p* < 0.05 for all time points from P50 in both LEH and SD rats). Further analysis revealed no significant difference between the strains (LME-ML: F_(1, 27)_ = 1.468, *p* = 0.236) but a significant main effect of week (F_(5, 134)_ = 12.44, *p* < 0.0001), and no significant interaction between strain and week (F_(5, 134)_ = 1.957, *p* = 0.089). These data suggest that OPR memory emerges around seven weeks of age in both SD and LEH rats.

Analysis of total sample phase object exploration indicated no significant difference between the strains (Figure 4C; LME-ML: F_(1, 27)_ = 0.030, *p* = 0.863) but there was a significant main effect of week (F_(5, 134)_ = 3.193, *p* = 0.009), and a significant strain × week interaction (F_(5, 134)_ = 4.84, *p* < 0.001). However, *post-hoc* tests indicated that the strains did not differ significantly from one another at any time point. There was no main effect of strain (LME-ML: F_(1, 27)_ = 1.139, *p* = 0.295) or week (F_(5, 134)_ = 0.443, *p* = 0.818) for total test phase object exploration, but a significant strain × week interaction was found (Figure 4D; F_(5, 134)_ = 2.444, *p* = 0.037). *Post-hoc* tests indicated that the strains differed significantly in total test phase object exploration only on week nine, where SD animals explored objects significantly more than LEH. Correlation analyses for OPR revealed no significant correlations between DI and object exploration during either sample or test phase for SD or LEH rats (Supplementary Table 3). Therefore, there is no indication that the fluctuations in sample phase or test phase object exploration, which were both consistently high, can account for the emergence of significant OPR memory at 7 weeks.

### Object-place-context memory emerges around seven weeks of age

**FIGURE 5:**
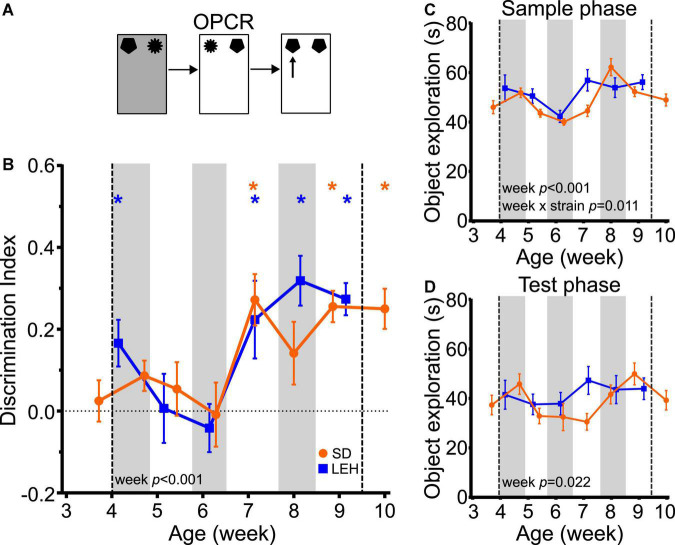
Object-place-context recognition memory emerges at around 7 weeks of age in LEH and SD rats. **(A)** Schematic of object-place-context recognition task; **(B)** Object-place-context recognition memory performance, expressed as discrimination index across development. Both LEH and SD rats show significant memory consistently from the 7th week of age (P50 for both LEH and SD), although LEH rats also show significant memory at 4 weeks of age (P39) before performing at chance on weeks five and six. *p < 0.05. A significant main effect of week was found; **(C)** Object exploration during the sample phases (mean of the two sample phases) for each testing time point for both LEH and SD rats. A significant main effect of week and a significant week × strain interaction were detected for mean object exploration during the sample phases; **(D)** Object exploration during the test phase. No significant main effects or interactions were detected. [SD]: *n* = 16 except P38&P50 where *n* = 15; [LEH]: *n* = 13 except P43&P57 where *n* = 12. For details on sample sizes, *t*, and *p*-values for one-sample *t*-tests, see Supplementary Table 2. Asterisks, shading on graphs etc., follow same convention as Figure 2.

Object-place-context recognition memory showed a very similar developmental time course as OPR memory. Discrimination ratios were significantly higher than chance (one-sample *t*-tests vs. chance levels *p* < 0.05) for all time points from P50 in LEH and SD rats except for P56 in SD rats (Figure 5B and Supplementary Figures 4D, 5D for individual animal data). Further analyses indicated that there was no significant difference between the strains (LME-ML: F_(1, 27)_ = 0.454, *p* = 0.506), but there was a significant main effect of week (F_(5, 131)_ = 6.7, *p* < 0.0001) with no significant strain × week interaction (F_(5, 131)_ = 0.921, *p* = 0.469). Thus, like OPR memory, OPCR memory emerges at around 7 weeks of age in both SD and LEH rats.

Analysis of total sample phase object exploration revealed no significant main effect of strain (Figure 5C; F_(1, 27)_ = 2.071, *p* = 0.162), but a significant main effect of week (F_(5, 131)_ = 9.906, *p* < 0.0001) and a significant strain × week interaction (F_(5, 131)_ = 3.128, *p* = 0.011). However, *post-hoc* testing indicated that there were no significant differences between strains at any time point, and both strains exhibited high levels of sample phase object exploration throughout testing. Total test phase object exploration also remained high throughout the experiment with no significant main effects of strain (F_(1, 27)_ = 0.831, *p* = 0.37), or week (F_(5, 131)_ = 2.73, *p* = 0.022), and no significant strain × week interaction (Figure 5D; F_(5, 131)_ = 1.75, *p* = 0.129). Correlation analyses revealed only a significant negative correlation between DI and test phase object exploration for SD rats (Supplementary Table 3; DI vs. Test: *R* = −0.286, *p* < 0.01), and no significant correlations between DI and object exploration for LEH. As discussed earlier, we have no reason to expect that a decrease in test phase object exploration would promote OPCR memory. Moreover, the similar developmental trajectory of OPCR memory in SD and LEH rats, and the absence of significant strain × week interaction in test phase object exploration suggest that the variation in test phase object exploration in SD rats is unlikely to be contributing to the developmental emergence of OPCR memory at 7 weeks. Taken together, these data indicate that OPCR memory emerges around the 7th week of age in both strains, and this is unlikely to be secondary to the fluctuations in object exploration in sample and test phases.

Overall, longitudinal testing revealed distinct developmental trajectories across the four spontaneous object exploration tasks in an albino and hooded strain (LME-ML, SD: task × age F_(15, 298)_ = 1.756, *p* = 0.040; LEH: F_(15, 234)_ = 1.848, *p* = 0.029). Object recognition memory emerged before four weeks of age, object-context memory emerged at around 5 weeks, while object-place and object-place-context memories emerged around 7 weeks of age.

Despite the spontaneous nature of object exploration tasks, it is plausible that repeated testing over juvenile development leads to context and object memory interference, as well as gradual context habituation, both of which could influence the developmental trajectory of memory in the different tasks. To address this possibility, we conducted a separate cross-sectional study, in which rats were tested at only one time point (age) on the four tasks. For this study we used both males and females from a different hooded rat strain (Lister Hooded–LH). LH rats have been shown to have very similar performance in visual and spatial tasks to LEH rats (Kumar et al., 2015).

### Cross sectional testing in Lister Hooded rats reveals similar developmental trajectories as longitudinal testing in Long Evans Hooded and Sprague Dawley rats

**FIGURE 6:**
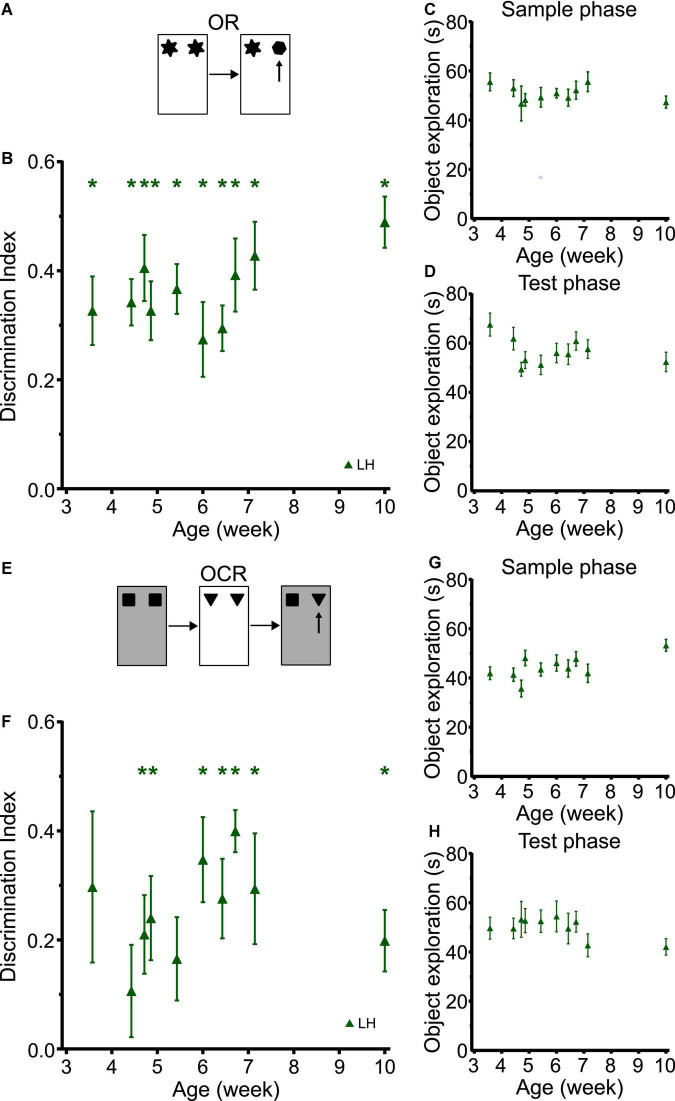
LH rats tested in a cross-sectional study design exhibit similar developmental trajectories for object and object-context memory to LEH and SD rats. **(A)** Schematic of object recognition task; **(B)** Object recognition memory performance, expressed as discrimination index, for different cohorts of rats tested at different ages. Green asterisks indicate significant difference from chance (DI = 0), based on one-sample *t*-tests. LH rats show significant object recognition memory from the first time tested (P25) *p < 0.05. No significant effect of age was detected; **(C)** Object exploration during sample phase. No significant effect of age was detected; **(D)** Object exploration during test phase. No significant effect of age was detected; **(E)** Schematic of object-context recognition task; **(F)** Discrimination indices for object-context recognition task. LH rats show significant object-context recognition memory from P33 onwards, except at P42; **(G)** Object exploration during the sample phases of the OCR task (mean of the two sample phases). A significant main effect of age was detected; **(H)** Object exploration during test phase of the OCR task. There were no significant differences across age. [OR]: P25 (n_(females)_ = 8, n_(males)_ = 10), P31 (n_(females)_ = 13, n_(males)_ = 14), P33 (n_(females)_ = 4, n_(males)_ = 4), P34 (n_(females)_ = 6, n_(males)_ = 10), P38 (n_(females)_ = 6, n_(males)_ = 9), P42 (n_(females)_ = 8, n_(males)_ = 8), P45 (n_(females)_ = 8, n_(males)_ = 9), P47 (n_(females)_ = 7, n_(males)_ = 9), P50 (n_(females)_ = 7, n_(males)_ = 8), P70 (n_(females)_ = 8, n_(males)_ = 8); [OCR]: P25 (n_(females)_ = 8, n_(males)_ = 8), P31 (n_(females)_ = 11, n_(males)_ = 13), P33 (n_(females)_ = 4, n_(males)_ = 4), P34 (n_(females)_ = 6, n_(males)_ = 9), P38 (n_(females)_ = 5, n_(males)_ = 9), P42 (n_(females)_ = 8, n_(males)_ = 8), P45 (n_(females)_ = 8, n_(males)_ = 9), P47 (n_(females)_ = 7, n_(males)_ = 7), P50 (n_(females)_ = 7, n_(males)_ = 8), P70 (n_(females)_ = 8, n_(males)_ = 8). For details on sample sizes, *t*, and *p*-values for one-sample *t*-tests, see Supplementary Table 2.

In the cross-sectional study with LH rats, object recognition memory was observed at the earliest time point tested (P25) which is similar to what we observed in the longitudinal studies in LEH and SD rats (Figure 6B and Supplementary Figure 6 shows individual animal data for all four tasks; strain: F_(2, 59)_ = 0.655, *p* = 0.523; strain × age: F_(8, 196)_ = 0.779, *p* = 0.622). LH rats had discrimination indices significantly better than chance at all time-points (one-sample *t*-tests vs. chance levels *p* < 0.05 for all time points), and there was no significant main effect of age (LME-ML: F_(9, 153)_ = 1.31, *p* = 0.238). Moreover, rats of all ages showed consistently high sample and test phase object exploration with no significant differences between rats tested at different ages either in sample phase object exploration (Figure 6C; LME-ML: F_(9, 120)_ = 0.678, *p* = 0.727) or in test phase object exploration (Figure 6D, F_(9, 120)_ = 1.656, *p* = 0.107).

The developmental trajectory of OCR memory for LH rats tested cross-sectionally was also similar to that found in SD and LEH rats tested longitudinally (Figure 6F; strain: F_(2, 59)_ = 1.098, *p* = 0.340; strain × age: F_(8, 194)_ = 0.320, *p* = 0.958). Rats tested at P25 and P31 did not show significant memory (*p* > 0.05), but from P33 rats exhibited above chance discrimination at all ages except for P38 (one-sample *t*-tests vs. chance levels *p* < 0.05 for all time points from P33 except P38). We did not find any significant main effect of age in the OCR discrimination ratios (LME-ML: F_(9, 111)_ = 1.12, *p* = 0. 356). Further analysis revealed no significant main effect of age in either the total sample phase object exploration (Figure 6G; LME-ML: F_(9, 111)_ = 1.96, *p* = 0.051), or the total test phase object exploration (Figure 6H; LME-ML: F_(9, 144)_ = 0.687, *p* = 0.719). The similar trajectories of OC memory in the longitudinal and cross-sectional studies suggest that exposure to multiple objects and repeated exposure to the contexts had negligible effect on developmental trajectories observed in the longitudinal studies.

**FIGURE 7:**
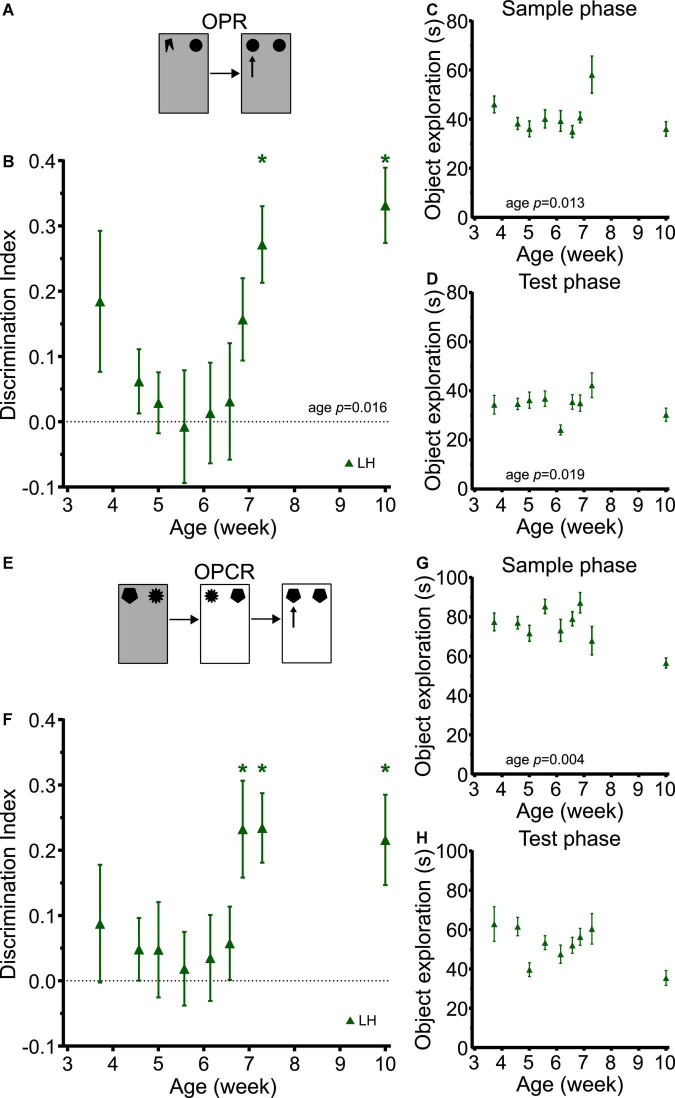
LH rats exhibit similar developmental trajectories for object-place and object-place-context memory to LEH and SD rats. **(A)** Schematic of object-place recognition task; **(B)** Object-place recognition memory performance, expressed as discrimination index for different cohorts of rats tested at different ages. LH rats show significant object place recognition memory only on P51 and P71. *p < 0.05. A significant main effect of age was found; **(C)** Object exploration during OPR sample phase. A significant main effect of age was detected; **(D)** Object exploration during test phase. A significant main effect of age was detected; **(E)** Schematic of object-place-context recognition task. **(F)** Discrimination indices for Object-place-context recognition memory performance. LH rats show significant object place context recognition memory only on P48, P51, and P71. No significant effect of age was detected; **(G)** Object exploration during OPCR sample phases (mean of the two sample phases). A significant main effect of age was detected; **(H)** Object exploration during OPCR test phase. A significant main effect of age was detected. [OPR]: P26 (n_(females)_ = 8, n_(males)_ = 9), P32 (n_(females)_ = 13, n_(males)_ = 14), P35 (n_(females)_ = 6, n_(males)_ = 9), P39 (n_(females)_ = 6, n_(males)_ = 10), P43 (n_(females)_ = 8, n_(males)_ = 8), P46 (n_(females)_ = 8, n_(males)_ = 9), P48 (n_(females)_ = 7, n_(males)_ = 9), P51 (n_(females)_ = 7, n_(males)_ = 8), P71 (n_(females)_ = 8, n_(males)_ = 8); [OPCR]: P26 (n_(females)_ = 8, n_(males)_ = 8), P32 (n_(females)_ = 13, n_(males)_ = 14), P35 (n_(females)_ = 6, n_(males)_ = 10), P39 (n_(females)_ = 6, n_(males)_ = 10), P43 (n_(females)_ = 6, n_(males)_ = 6), P46 (n_(females)_ = 8, n_(males)_ = 8), P48 (n_(females)_ = 7, n_(males)_ = 8), P51 (n_(females)_ = 6, n_(males)_ = 6), P71 (n_(females)_ = 7, n_(males)_ = 8). For details on sample sizes, *t*, and *p*-values for one-sample *t*-tests, see Supplementary Table 2.

Object-place recognition memory showed a clear developmental trajectory (Figure 7B). Despite the differences in study design and rat strain, this trajectory was very similar to those in SD and LEH rats in the longitudinal study (strain: F_(2, 252)_ = 1.284, *p* = 0.279; strain × age: F_(8, 252)_ = 1.009, *p* = 0.429), with LH rats exhibiting discrimination indices significantly above chance levels from P51 (just over 7 weeks old) but not at earlier time points (Figure 7B; one-sample *t*-tests vs. chance levels *p* < 0.05 for all ages from P51). A significant main effect of age was found for OPR memory (LME-ML: F_(8, 115)_ = 2.48, *p* = 0.016). The sample phase and test phase object exploration was high throughout the experiment (>20 s all time points) (Figures 7C,D). However, our analysis revealed a significant main effect of age on object exploration during both the sample phase (LME-ML: F_(8, 115)_ = 2.567, *p* = 0.013) and the test phase (LME-ML: F_(8, 115)_ = 2.42, *p* = 0.019).

Correlation analyses revealed a significant positive correlation between DI and sample phase object exploration (Supplementary Table 3; DI vs. Sample: *R* = 0.173, *p* = 0.031). This raises the possibility that increased sample phase exploration in LH rats may contribute, at least in part, to the OPR performance at some ages. From inspection of the sample phase object exploration data, it appears that exploration in the rats tested at P51 was higher than that of rats tested at earlier time points, and this is the first day on which significant OP memory was observed. However, we think it unlikely that this can account for the emergence of OP memory for two reasons. First, the group tested on P71 showed comparable sample phase object exploration as those tested at earlier time points (i.e., before OP memory was observed), yet still showed significant OP memory. Second, there was no significant correlation between sample phase object exploration and OP memory in the longitudinal studies with SD and LEH rats, where the time course for the emergence of OPR memory was identical. Therefore, it is more parsimonious to conclude that OP memory emerges at around the same point in the three strains of rats independent of sample phase object exploration fluctuations, rather than proposing that in the LH rats it is due to increased object exploration, but that in the LEH and SD rats it is due to some other variable.

The developmental trajectory of OPCR memory in the LH rats tested cross-sectionally was also very similar to those in SD and LEH rats in the longitudinal study (strain: F_(2, 244)_ = 0.104, *p* = 0.902; strain × age: F_(8, 244)_ = 10.517, *p* = 0.843), with LH discrimination indices significantly above chance levels from P48 (7 weeks old) but not at earlier time points (Figure 7F; one-sample *t*-tests vs. chance levels *p* < 0.05 for all ages from P48 in OPCR). No significant main effect of age was detected for OPC discrimination performance (LME-ML: F_(8, 105)_ = 1.77, *p* = 0.092). The total object exploration during testing was high for all rat ages. However, our analysis revealed a significant main effect of age on object exploration during both the sample phase (Figure 7G; LME-ML: F_(8, 105)_ = 3.08, *p* = 0.004) and test phase (Figure 7H; LME-ML: F_(8, 105)_ = 1.826, *p* = 0.080). Correlation analyses revealed no significant correlations between DI and sample or test phase exploration. Overall, these analyses suggest that the emergence of memory for object-place-context associations at around 7 weeks of age is not due to changes in object exploration during the sample or test phases.

For the cross-sectional study we used both female and male LH rats. Importantly, our analyses revealed no significant differences between sexes or significant age × sex interactions for DIs any of the four tasks [2-way ANOVA (OR): Sex: F_(1, 143)_ = 1.274, *p* = 0.277; Sex-Age interaction: F_(9, 143)_ = 0.893, *p* = 0.534; (OCR): Sex: F_(1, 134)_ = 1. 790, *p* = 0.183; Sex-Age interaction: F_(9, 134)_ = 0.427, *p* = 0.919; (OPR): Sex: F_(1, 137)_ = 2.738, *p* = 0.10; Sex-Age interaction: F_(8, 137)_ = 1.928, *p* = 0.061; (OPCR): Sex: F_(1, 127)_ = 0.109, *p* = 0.742; Sex-Age interaction: F_(8, 127)_ = 1.195, *p* = 0.307].

Finally, in addition to our main statistical analyses in which litter identity was fitted as random factor, we performed a more stringent set of analyses in which discrimination indices were averaged across all rats from the same litter (SD & LEH) in the longitudinal study, or across all rats of the same sex from the same litter in the cross sectional study (LH) (Supplementary Figures 7–9). This was to ensure maximum control for intra-litter correlations. While these analyses were underpowered, the results were largely consistent with those of our main analyses. Litters performed above chance at all ages in OR, while OPR and OPCR memory emerged after 7th week for all three strains. The developmental trajectory of OCR memory was the most inconsistent with our previous analyses, with SD litters performing above chance levels after 5 weeks, LEH after 6 weeks and LH after 4 weeks of age.

## Discussion

We investigated the ontogeny of episodic-like object-place-context memory and of memory in three tasks requiring memory for objects, object-context and object-place associations in rats. We found that three different outbred rat strains that are commonly used in basic neuroscientific and neuropsychiatric research exhibit remarkably similar developmental trajectories in their ability to recognize objects and object-context, object-place and object-place-context associations. Moreover, the trajectories for the different tasks are distinct. This likely reflects the development of the distinct neural circuits needed to support encoding and/or retrieval of memory in the different tasks. Interestingly, the developmental trajectories were unaffected by study design (i.e., longitudinal or cross-sectional), suggesting that repeated exposure to objects and contexts did not affect the developmental trajectory of object, object-context, object-place or object-place-context memory in the current study. This work adds to a large body of literature on the developmental trajectories of cognition in rodents (Hunt et al., 2016; Tan et al., 2017; Donato et al., 2021).

Here we report that rats were able to recognize objects from the first testing time point (3–4 weeks old depending on strain) in our studies (Figures 2, 6). This is consistent with our previous work and findings from other labs showing that rats exhibit object memory as early as two-weeks old (Ainge and Langston, 2012; Krüger et al., 2012; Jablonski et al., 2013; Westbrook et al., 2014; Cruz-Sanchez et al., 2020). The perirhinal cortex is generally agreed to be required for object recognition memory whereas the entorhinal cortex, hippocampus (HPC), and medial prefrontal cortex (mPFC) are not (although there is debate concerning the role of the HPC at longer retention intervals and its normal role in the intact brain) (Dix and Aggleton, 1999; Brown and Aggleton, 2001; Eacott and Norman, 2004; Norman and Eacott, 2005; Langston et al., 2010; Wilson et al., 2013a,b; Chao et al., 2016). Although, the postnatal development of rat perirhinal cortex has not been fully characterized, morphological analysis of rat perirhinal neurons between birth and late adolescence (P45) suggests that perirhinal cortex may be fully developed around the time of eye-opening (P12–15) (Furtak et al., 2007). This would be consistent with OR memory (at least for relatively short retention intervals) developing at around this age. Overall, the demonstration that object recognition memory is established early in juvenile development is fundamental for the interpretation of findings from more complex types of memory involving associations between objects and spatio-temporal features of the environment. Being able to remember object identities at all experimental time points suggests that the different developmental trajectories observed for the other three tasks are not due to inability to distinguish novel from familiar objects.

In contrast to object memory, the ability to recognise object-context associations did not emerge until around 5 weeks of age in all three rat strains (Figures 3, 6). Our data appear to be at odds with previous work from Ramsaran et al. (2016a,b), who showed that object-context recognition memory emerges during the second week of life in LEH rats (Ramsaran et al., 2016b). However, there are some crucial methodological differences between our work and that of Ramsaran et al. (2016b). The differences are centered on the nature of the contextual information. When contexts differ in testing arena wall and floor colour and texture as well as in polarising intra-maze cues, and the arenas are in different experimental rooms providing different distal spatial cues, rats can detect novel object-context association from two weeks old (Ramsaran et al., 2016b). If the two contexts do not differ in local contextual information but the two testing arenas are situated in different experimental rooms with different distal spatial information, OCR memory emerges during the third postnatal week (Ramsaran et al., 2016b). In our experiments, contexts were defined only by floor and wall inserts with different texture and color, while intra-maze and distal cues conferring polarising spatial information, as well as the position of the arena within the room, remained the same between testing phases. Therefore, the contextual differences in our experiments are very different than those in the Ramsaran et al studies. It is plausible that the nature of the contextual differences (i.e., intra-maze contextual information, prominent directional cues and spatial frame, spatial frame only, intra-maze only) may be a key determinant of the neural circuits that are required to support OCR memory. The differing developmental trajectories observed may therefore reflect different development of these circuits. Alternatively, the differences between the studies could be explained by the fact that in the studies by Ramsaran et al. (2016a,b), rats were tested only once in a single task (OCR), whereas the rats in our study had been tested on an OR task earlier on the same day. Therefore, object or context interference from OR testing in the same day could have affected the ontogeny of OCR memory in our study.

Using a very similar protocol to one used in our studies, where context is based only on local contextual cues (floor and walls) but all distal and polarising spatial information is kept constant, it has been demonstrated that the lateral entorhinal cortex (LEC) is necessary for object-context memory (Wilson et al., 2013a,b), as is postrhinal cortex (Norman and Eacott, 2005), but not HPC (Langston and Wood, 2009), or fan cell inputs from LEC to HPC (Vandrey et al., 2020). However, when contextual differences involve more salient changes in spatial frames (e.g., different testing room, different distal cues, or different arena geometry and polarising intra-maze cues) then HPC is necessary (Balderas et al., 2008; Barker and Warburton, 2020). In addition to postrhinal cortex, LEC and HPC, the medial entorhinal cortex (MEC) could also be involved in object-context associative memory, as it has been shown to be essential for detection of contextual novelty such as texture and colour in floor and wall of an enclosure as well as convey contextual information to the hippocampus (Hunsaker et al., 2013; Kitamura et al., 2015).

Taken together, the present evidence suggests that postrhinal cortex, LEC, and possibly MEC (but not HPC) play a key role in associating contexts with objects when the contexts can be discriminated only on the basis of local non-spatial intra-maze cues. This leads to the hypothesis that this circuitry may not develop sufficiently to support OC memory until around five weeks of age. While the postnatal development of LEC is largely unknown, *in vivo* electrophysiological studies by us and others suggest that the functional maturation of spatial firing of MEC neurons may be complete by around five weeks, consistent with this time frame for OCR emergence (Langston et al., 2010; Wills et al., 2010). In contrast, when contexts can be discriminated on the basis of distal spatial information and/or geometric/polarising changes to the environment, then the hippocampus and its interactions with MEC may play a more prominent role in object-context memory (Shan et al., 2022). Consistent with this proposal, the spatial information contained in place cell firing as well as the stability of spatial representations between exposures in the same environment are similar to adult levels by four weeks of age (Langston et al., 2010; Wills et al., 2010; Farooq and Dragoi, 2019).

Memory in the OPR task requires the binding of location within the environment and object identity information and is known to depend on the coordination of a number of intact brain circuits, including the LEC and its connections to mPFC (Wilson et al., 2013b; Chao et al., 2016). Memory in this version of the task does not require the hippocampus, at least at the short retention intervals used in the current study (Langston and Wood, 2009). We suggest that the functional maturation of LEC-mPFC circuits may be dictating the developmental trajectory of OPR as explained in our discussion of OPCR memory below. The trajectory we observed in the OPR task differs from that previously reported for the more commonly used Object Location (OL) task, which tests the ability to detect that an object has moved to a novel location rather than memory for associations between specific objects and their locations. The ability to detect spatial novelty in the OL task has been reported to be in place by three weeks old (Krüger et al., 2012; Jablonski et al., 2013). As OL recognition does not require LEC or mPFC (Chao et al., 2016) but instead requires an intact hippocampus (Barker and Warburton, 2011), the developmental emergence of OL memory is consistent with the early development of the hippocampus described above.

The late emergence of OPR memory in the current study contradicts our previous findings that OPR memory is in place shortly after 4 weeks of age (Ainge and Langston, 2012). At face value this is an unexplained result. However, our previous work involved cross-sectional testing in only OR and OPR memory tasks which took place in a single context. Therefore, rats were not exposed to multiple contexts during habituation and during OCR testing before OPR. It is plausible that the current experimental design using 2 contexts leads to more memory interference during any two-day testing time point.

The late development of episodic-like OPCR memory in rats is generally consistent with the developmental trajectory of episodic memory in humans (Guillery-Girard et al., 2013; Riggins et al., 2020; Ngo et al., 2021). OPCR memory requires an intact HPC and LEC as well as LEC-HPC, LEC-mPFC, and HPC-PFC interactions (Langston and Wood, 2009; Wilson et al., 2013b; Chao et al., 2016; Barker and Warburton, 2020; Vandrey et al., 2020). Given that OPR and OPCR appear to have a very similar developmental trajectory, which is distinct from the LEC-dependent OCR trajectory, it is unlikely that LEC circuit maturation controls the emergence of OPR and OPCR memory (Figures 4, 5, 7).

We propose that the late emergence of both OPCR and OPR memory may be dictated by the time course of mPFC circuit maturation. This developmental trajectory is consistent with previous work showing that young adolescent rats (<P39) are worse at attentional set-shifting and are more impulsive compared to young adults (>P66) (Newman and McGaughy, 2011; Doremus-Fitzwater et al., 2012). Both impulsivity and attention set-shifting behaviour have been linked to prefrontal function (Birrell and Brown, 2000; Bradshaw et al., 2016).

While several aspects of mPFC circuit function develop early in postnatal development (Chini and Hanganu-Opatz, 2021) others mature much later during adolescence in rats (Caballero et al., 2016) and even during adulthood in mice (Mukherjee et al., 2019). More specifically, local inhibitory networks within mPFC that allow gating of HPC inputs do not develop fully until the seventh postnatal week in rats (Tseng and O’Donnell, 2007; Caballero et al., 2016; Caballero and Tseng, 2016). While the developmental trajectory of LEC-mPFC interactions is not known, it is plausible that the protracted development of mPFC inhibition determines the functionality of both LEC-mPFC and HPC-mPFC interactions and ultimately the developmental trajectory of OPR and OPCR memory.

A previous study has reported that object-place-context recognition memory is in place during the fifth postnatal week (Ramsaran et al., 2016a) which appears to contradict our current findings. However, as discussed above, the contexts used in the experiments by Ramsaran et al. (2016a) consisted of radically different testing enclosures situated in different experimental rooms, which may be coded by MEC-HPC. Therefore, OPCR memory in which contexts can be defined on the basis of distal spatial information may rely on different circuits that OPCR memory that requires binding of object and place information with non-polarising intra-maze contextual cues. We suggest that the LEC and its interactions with both HPC and mPFC may only be required in the latter case. An alternative explanation for the different findings between our study and Ramsaran et al., with regards to the age of emergence of OPCR memory, may be that rats in our study were tested on OR, OCR, and OPR prior to OPCR, leading to memory and contextual interference. Future experiments in which rats are tested in a single task (either OCR, OPR, or OPCR) at just one time point will allow us to test whether previous testing within each time-point leads to interference, thereby delaying the ontogeny of these abilities.

The current findings allow us to formulate two testable hypotheses. The first is that local LEC circuit function matures around the fifth postnatal week to support object-context recognition memory. The second is that mPFC circuit function, more specifically the inhibitory control of inputs from both LEC and HPC, matures around the seventh postnatal week to support object-place and object-place-context memory. One advantage of rat models compared to mice is the ability to use *in vivo* electrophysiology to study circuit function during juvenile development (Langston et al., 2010; Wills et al., 2010; Farooq and Dragoi, 2019). Therefore, an obvious future direction from our findings is to explore the developmental trajectory of circuit functions in relation to the developmental trajectories of object-context, object-place and episodic-like object-place-context memory.

The developmental trajectories we report here raise questions about the ontogeny of other associative object recognition memory tasks that assess aspects of episodic-like memory. For example the what-where-when (WWWhen) task that requires subjects to associate object identity, object location and temporal order/recency of object exposure (Kart-Teke et al., 2006). Similar to OPC (WWWhich) memory, WWWhen memory requires HPC and mPFC-LEC interactions (Chao et al., 2016; Drieskens et al., 2017; de Souza et al., 2019). Interestingly though, WWWhen and WWWhich memory have been shown to be differently affected by normal ageing and neurodegeneration (Davis et al., 2013), suggesting that the neural circuits mediating these tasks may differ in some way. It would be interesting to test whether the developmental trajectory of WWWhen and the WWWhich (OPCR) memory follow similar developmental trajectories.

Despite the overwhelming similarities in the developmental trajectories of the three rat strains in our study, there are some small differences, with the most usual being in the amount of exploration different strains exhibited in sample or/and test phase. This could reflect known differences in vision between albino and pigmented rat strains (Andrews et al., 1995; Prusky et al., 2002; Kumar et al., 2015; Waite et al., 2021). Despite the differences in object exploration between rat strains, our correlation analyses did not reveal any consistent relationships. The occasional significant correlations between total object exploration in the sample or test phases and discrimination performance varied between being negative and positive, and were strain specific (Supplementary Table 3). It has previously been argued that ensuring a minimum amount of object exploration during sampling phase is important for the interpretation of discrimination performance data (Cohen and Stackman, 2015). In our studies rats exhibited high levels of sample phase exploration throughout age points and tasks. Collectively, these data suggest that the developmental trajectories described here are not secondary to fluctuations in sample (or test) phase object exploration behaviour.

While our study was not designed to address the importance of sex as a determinant of object recognition memory development, we were able to explore this question in our cross-sectional experiment with LH rats. The absence of sex-dependent developmental trajectories in LH rats is consistent with recent research and meta-analyses suggesting that sex is not a significant determinant of object memory performance (Becker et al., 2016; Becegato and Silva, 2022).

Shared genetics and maternal environment in multiparous species can lead to high similarity between outcome variables in littermates that violate statistical independence. The most appropriate method to address intra-litter correlations and litter oversampling is considered to be the use of linear mixed effects models with litter included as a random effect (Golub and Sobin, 2020). Traditionally, the most stringent statistical approach has been to use one animal per litter or to average across animals from a litter, such that litter is the experimental unit. Here, both statistical analysis approaches for analysing the developmental trajectory of memory in SD and LEH rats have led to similar results.

Data from the cross-sectional study originated from rats coming from only 2–3 litters for each age group, with all animals from any given litter being assigned to a single age group. This experimental design poses some statistical challenges. Given the known intra-litter statistical dependencies, we have inadvertently oversampled from each litter. Taking advantage of the fact that we used both female and male rats in this study, we fitted linear mixed effects models with sex-within-litter as a nested random effect. This approach, similar to the one used in the longitudinal studies, can begin to account for intra-litter statistical dependencies. Our supplemental analyses on data averaged across rats of the same sex from the same litter (sex-within-litter as statistical unit), while underpowered, yielded very similar results to our main analyses.

An alternative approach for the cross-sectional study would have been to assign different littermates to different age groups, resulting in a quasi-repeated study design (i.e., same litter across multiple age groups). However, this approach presents its own unique limitations. The different duration between weaning and testing for littermates can yield distinct experiential contributions to behaviour, and complicate the effects of intra-litter correlations. Therefore, sampling for cross-sectional studies is particularly challenging when attempting to reduce the number of experimental subjects used.

Taken these considerations into account, and the fact that the developmental trajectories of OR, OCR, OPR, and OPCR memory in SD, LEH, and LH each mirror other, we suggest that it is extremely unlikely that the developmental trajectories we report here can be accounted for by our sampling methods.

Genetic rat models of neurodevelopmental conditions are providing new insights into behavioural and circuit abnormalities associated with mutations in genes of interest (Till et al., 2015; Asiminas et al., 2019; Berg et al., 2021). In order to understand the neural and circuit pathophysiology associated with cognitive deficits that emerge during postnatal development, and to identify key time points and targets for therapeutic intervention, it is important to utilize these rat models (Asiminas et al., 2019). More specifically, episodic-like memory tasks can offer good face validity since episodic memory is affected in neurodevelopmental conditions such autism and schizophrenia (Wang et al., 2010; Ragland et al., 2015; Cooper and Simons, 2019). The differential development of different types of associative recognition memory offers a unique opportunity to delineate the developmental trajectory and function of neural circuits supporting the different components of episodic memory. Using spontaneous object exploration tasks to explore deviations from normal developmental trajectories in specific tasks can provide a window to the circuit pathophysiology and progression of neurodevelopmental conditions.

## Data availability statement

The original contributions presented in this study are included in the article/Supplementary material, further inquiries can be directed to the corresponding authors.

## Ethics statement

This animal study was reviewed and approved by University of Edinburgh and University of Dundee Animal Welfare and Ethical Review Boards.

## Author contributions

AA, SL, RL, and EW conceived and designed the experiments. AA and SL collected and analysed the data. AA wrote the first draft manuscript. AA, RL, and EW interpreted all the results and wrote the manuscript. All authors contributed to the article and approved the submitted version.
