# Supplementary material for: Developmental trajectory of episodic-like memory in rats
Source: Front Behav Neurosci. 2022 Nov 29;16:969871. doi: 10.3389/fnbeh.2022.969871 (PMC9745197; doi:10.3389/fnbeh.2022.969871)
Supplement: Supplementary file 1 [file Data_Sheet_1.zip › Figure 1.PDF]

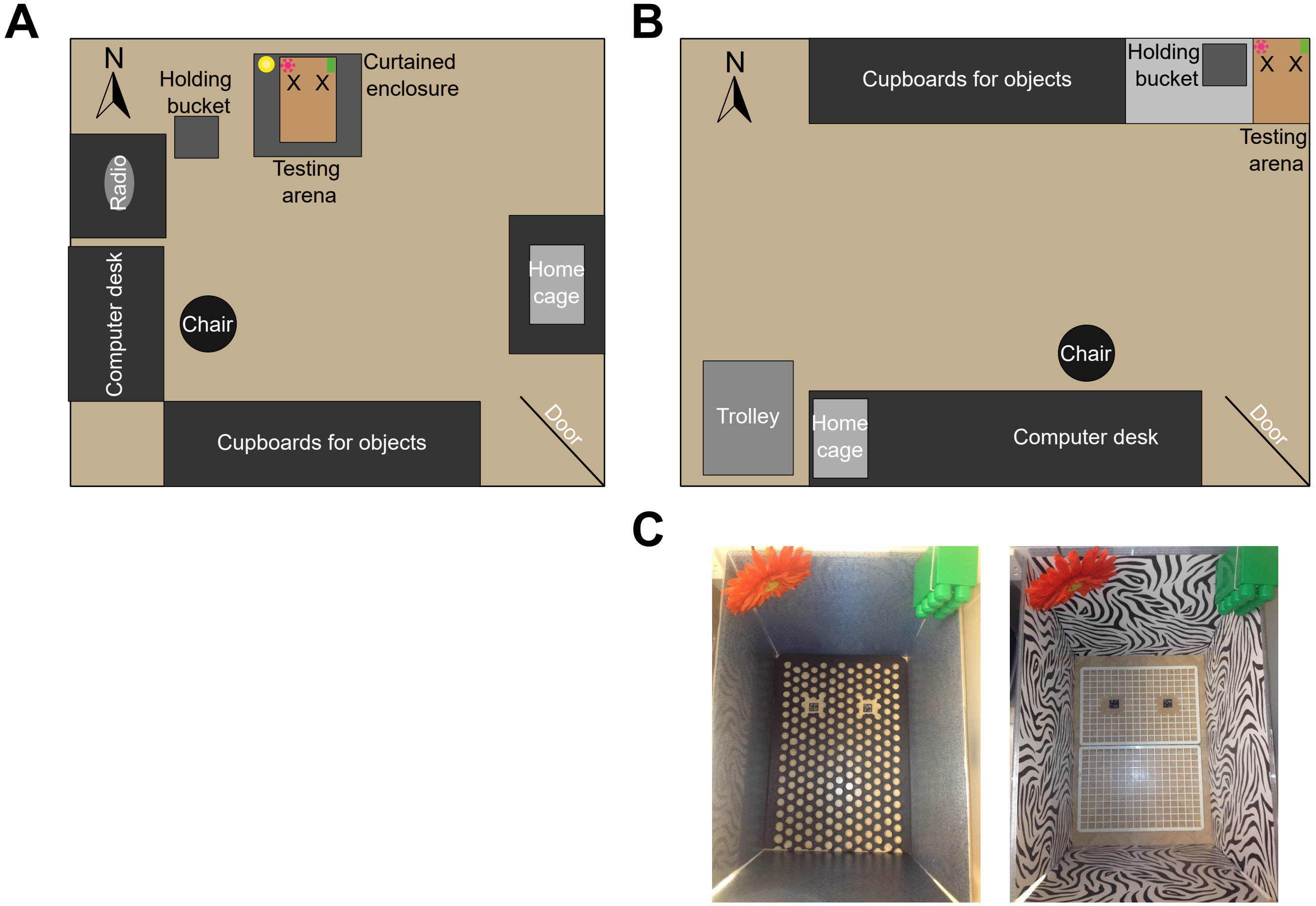

Supplementary Figure 1. (A) Schematic of the experimental room in Wood lab. The computer desk where the experimenter sat was positioned within 2 meters from the recording arena (brown box). A radio was used to mask potentially distracting noises. The holding bucket was placed next to the arena. A lamp (yellow circle) in the north-west corner of the curtained enclosure as well as two prominent cues on the north-west and north-east corner of the testing arena (red and green) remained in the same position and orientation throughout the experiments. The crosses represent object location left and right as viewed from above. The home cage containing the cage mates of the rat being tested was placed approximately 2 meters south west from the testing arena. **(B)** Schematic showing the layout of the experimental room in Langston lab. The testing arena is shown in the north-east corner of the room. The trolley used to transport the animals, along with the home cage of the animals undergoing testing, was placed in the corner furthest from the testing arena. **(C)** Photographs of the testing arena in Langston lab. Context 1 (left) has blue walls with a rubber mat floor, context 2 (right) has black and white walls with white plastic grid floor. The two prominent directional cues (flower and green lego block) were the same for both contexts.
