# Supplementary material for: Developmental trajectory of episodic-like memory in rats
Source: Front Behav Neurosci. 2022 Nov 29;16:969871. doi: 10.3389/fnbeh.2022.969871 (PMC9745197; doi:10.3389/fnbeh.2022.969871)
Supplement: Supplementary file 1 [file Data_Sheet_1.zip › Figure 2.PDF]

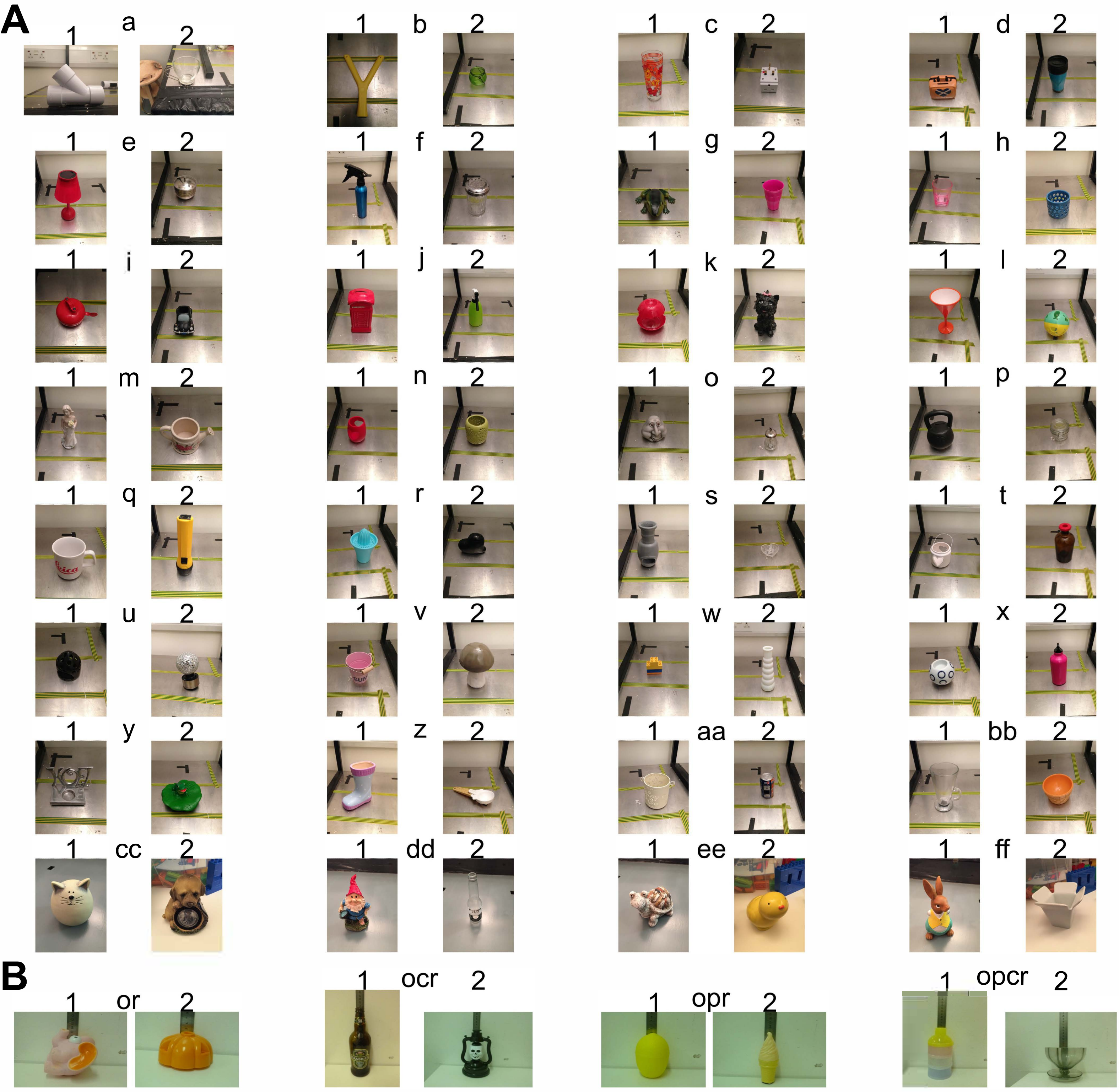

Supplementary Figure 2. List of object pairs used. (A) The object pairs used in longitudinal studies with Sprague-Dawley and Long-Evans Hooded rats. **(B)** The object pairs used in the cross-sectional study with Lister Hooded rats.
