# Supplementary material for: Developmental trajectory of episodic-like memory in rats
Source: Front Behav Neurosci. 2022 Nov 29;16:969871. doi: 10.3389/fnbeh.2022.969871 (PMC9745197; doi:10.3389/fnbeh.2022.969871)
Supplement: Supplementary file 1 [file Data_Sheet_1.zip › Figure 3.PDF]

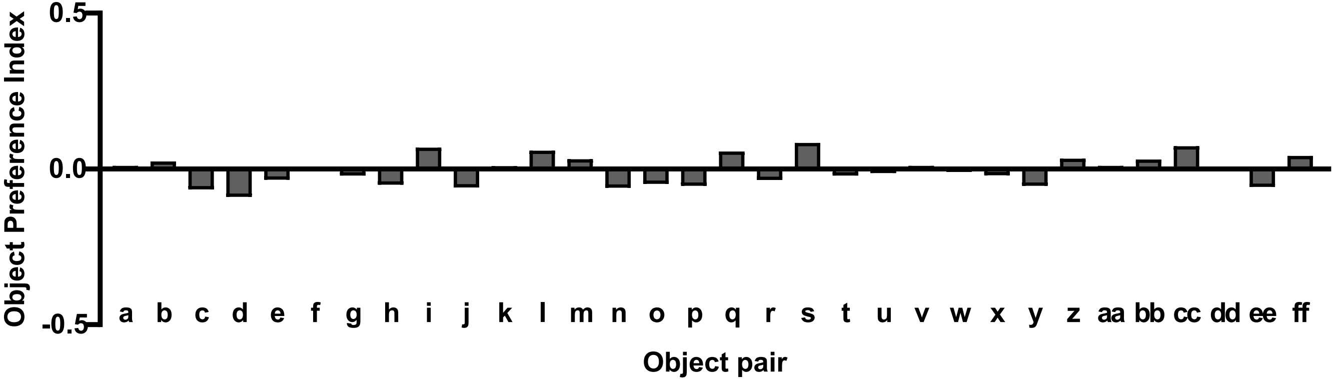

Supplementary Figure 3. Object preference for all object pairs used in the longitudinal studies. Object preference index for each object pair was calculated based on the average exploration for object 1 and object 2 of the pair. The sample explorations were pooled from across all tasks, rats and time-points a given object was used. Object preference index = (Mean exploration(object 1) - Mean exploration(object2))/ (Mean exploration(object 1) + Mean exploration(object2))
