# Supplementary material for: Developmental trajectory of episodic-like memory in rats
Source: Front Behav Neurosci. 2022 Nov 29;16:969871. doi: 10.3389/fnbeh.2022.969871 (PMC9745197; doi:10.3389/fnbeh.2022.969871)
Supplement: Supplementary file 1 [file Data_Sheet_1.zip › Figure 5.PDF]

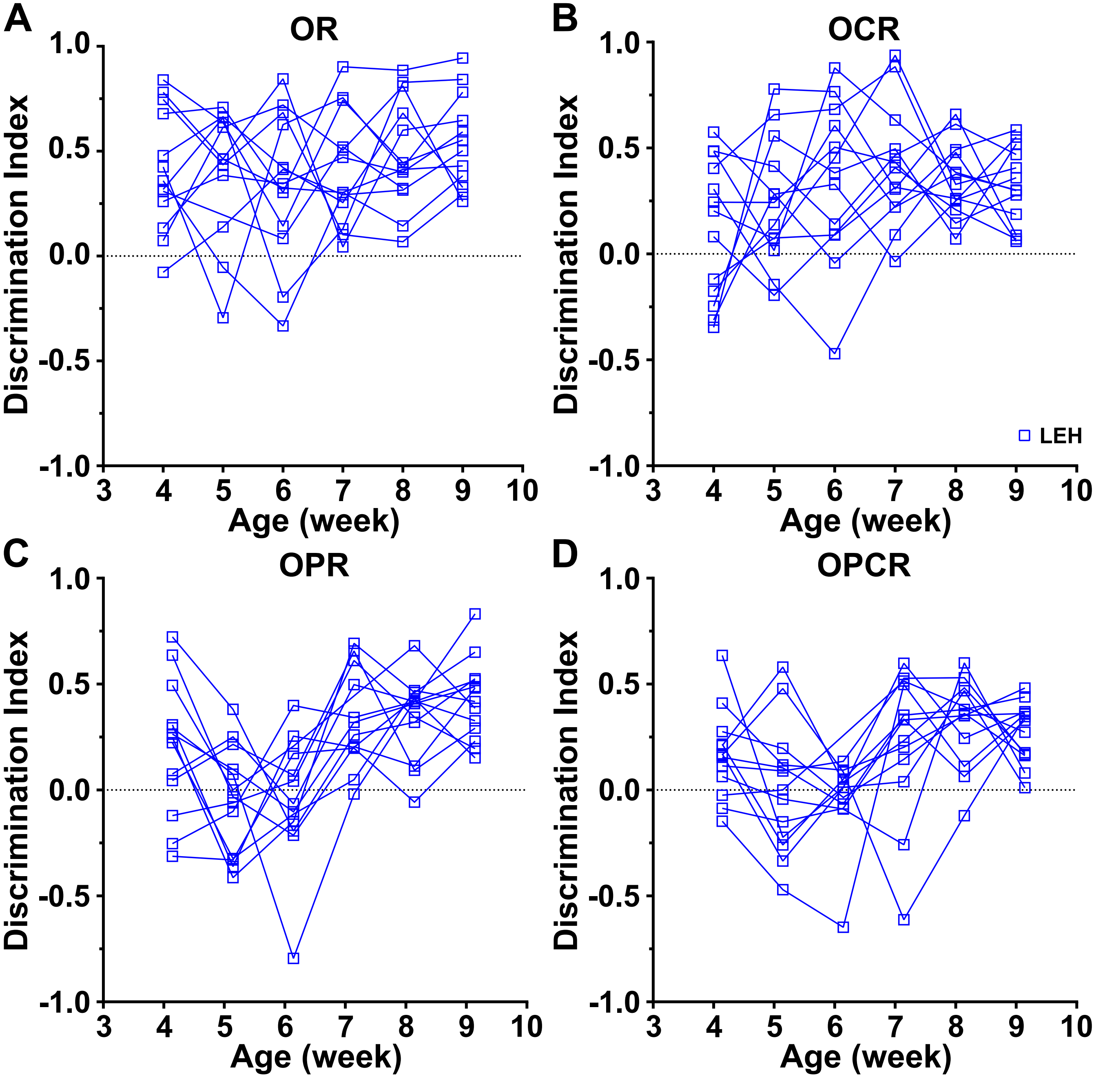

Supplementary Figure 5. (A-D) Individual discrimination index values for each LEH rat tested longitudinally in each task.
