# Supplementary material for: Developmental trajectory of episodic-like memory in rats
Source: Front Behav Neurosci. 2022 Nov 29;16:969871. doi: 10.3389/fnbeh.2022.969871 (PMC9745197; doi:10.3389/fnbeh.2022.969871)
Supplement: Supplementary file 1 [file Data_Sheet_1.zip › Figure 8.PDF]

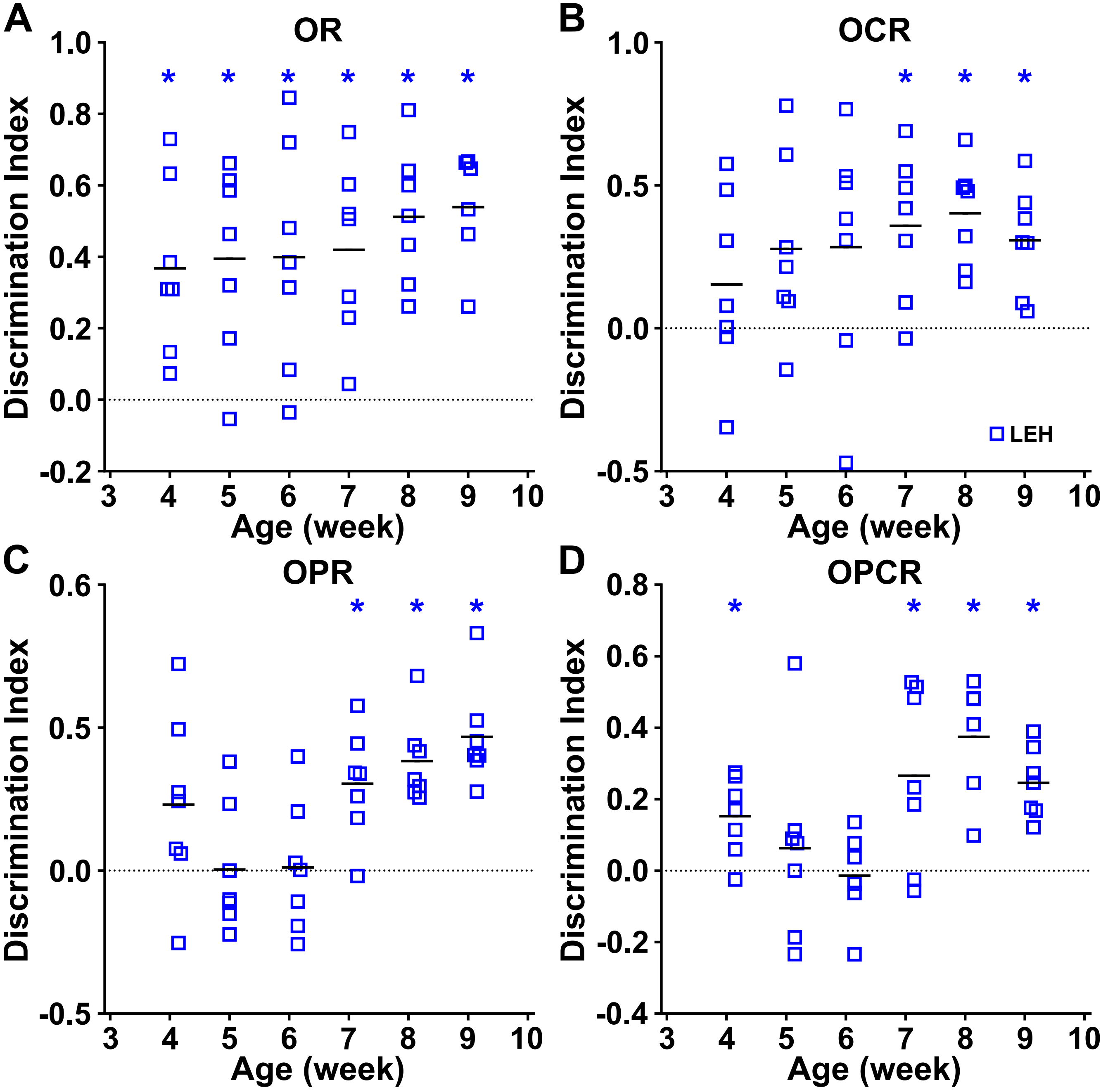

Supplementary Figure 8. Litter-average analysis for the developmental trajectory of OR, OCR, OPR and OPCR memory in LEH rats (A-D). Average discrimination index data across rats from the same litter, for each task. Individual points indicate values for specific litters. Horizontal black bars indicate mean values across all litters. Asterisks indicate significant difference from chance (DI =0) based on one-sample *t* tests. *$p < 0.05$. n=7 litters for all time points.
