# Supplementary material for: Developmental trajectory of episodic-like memory in rats
Source: Front Behav Neurosci. 2022 Nov 29;16:969871. doi: 10.3389/fnbeh.2022.969871 (PMC9745197; doi:10.3389/fnbeh.2022.969871)
Supplement: Supplementary file 1 [file Data_Sheet_1.zip › Figure 9.PDF]

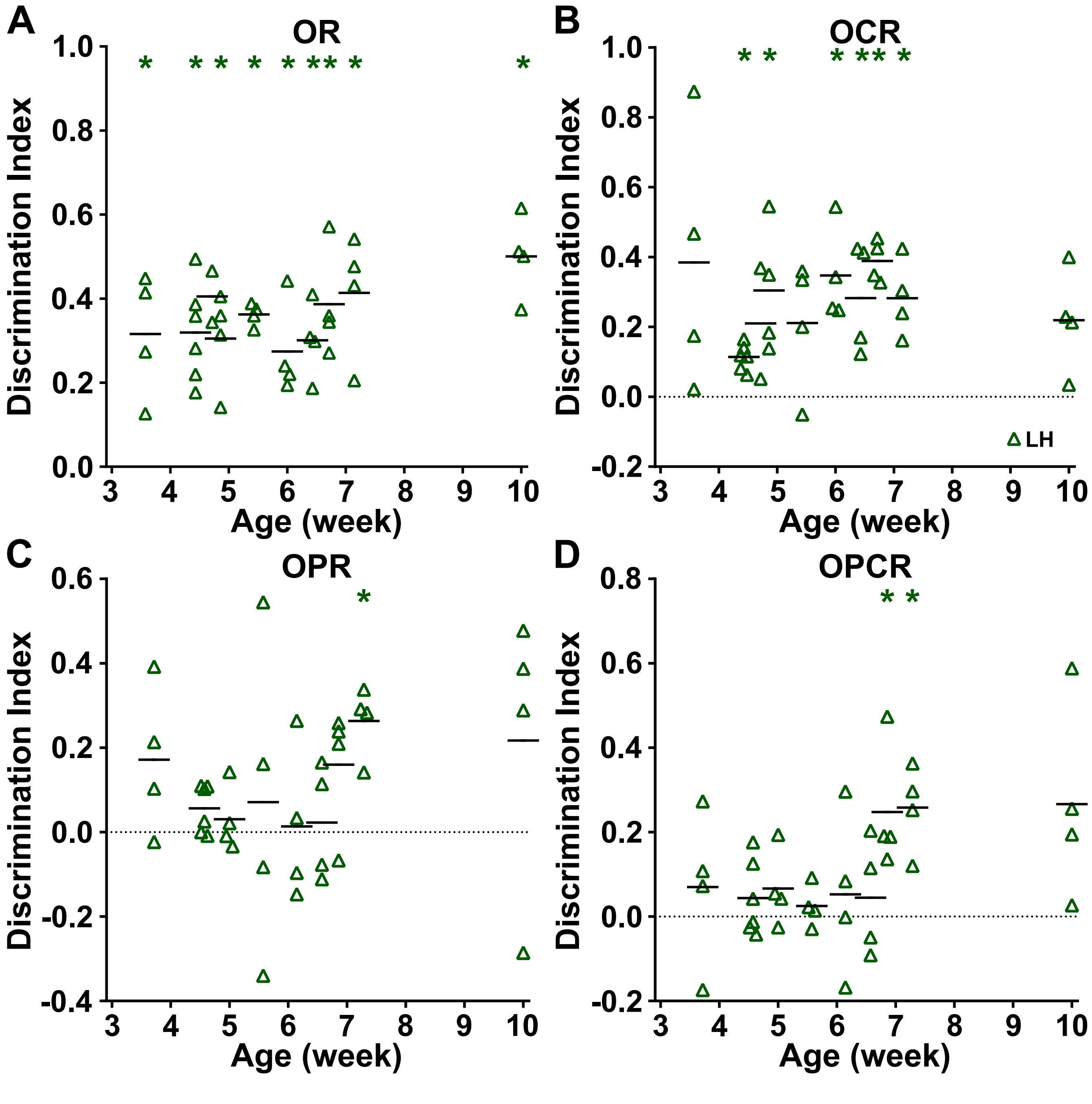

Supplementary Figure 8. Sex-within-litter-average analysis for OR, OCR, OPR and OPCR memory in LH rats (A-D). Average discrimination index data across rats of the same sex from the same litter, for each task. Individual points indicate values for specific sex-litters combinations. Horizontal black bars indicate mean values across all sex-litter combinations. Asterisks indicate significant difference from chance (DI =0) based on one-sample *t* tests. $*p < 0.05$. n=4 unique sex-litter combinations for all time points except from P32/33: n=6, and P34: n=2.
